# Proliferation of *Ty3/gypsy*-like retrotransposons in hybrid sunflower taxa inferred from phylogenetic data

**DOI:** 10.1186/1741-7007-7-40

**Published:** 2009-07-14

**Authors:** Mark C Ungerer, Suzanne C Strakosh, Kaitlin M Stimpson

**Affiliations:** 1 Division of Biology, Kansas State University, Manhattan, Kansas, USA; 2 Department of Molecular Genetics and Microbiology, Duke University, Durham, North Carolina, USA

## Abstract

**Background:**

Long terminal repeat (LTR) retrotransposons are a class of mobile genetic element capable of autonomous transposition via an RNA intermediate. Their large size and proliferative ability make them important contributors to genome size evolution, especially in plants, where they can reach exceptionally high copy numbers and contribute substantially to variation in genome size even among closely related taxa. Using a phylogenetic approach, we characterize dynamics of proliferation events of *Ty3/gypsy*-like LTR retrotransposons that led to massive genomic expansion in three *Helianthus *(sunflower) species of ancient hybrid origin. The three hybrid species are independently derived from the same two parental species, offering a unique opportunity to explore patterns of retrotransposon proliferation in light of reticulate evolutionary events in this species group.

**Results:**

We demonstrate that *Ty3/gypsy*-like retrotransposons exist as multiple well supported sublineages in both the parental and hybrid derivative species and that the same element sublineage served as the source lineage of proliferation in each hybrid species' genome. This inference is based on patterns of species-specific element numerical abundance within different phylogenetic sublineages as well as through signals of proliferation events present in the distributions of element divergence values. Employing methods to date paralogous sequences within a genome, proliferation events in the hybrid species' genomes are estimated to have occurred approximately 0.5 to 1 million years ago.

**Conclusion:**

Proliferation of the same retrotransposon major sublineage in each hybrid species indicates that similar dynamics of element derepression and amplification likely occurred in each hybrid taxon during their formation. Temporal estimates of these proliferation events suggest an earlier origin for these hybrid species than previously supposed.

## Background

The genomes of flowering plants are remarkably variable in nuclear DNA content, with >1,000-fold differences among some taxa [1,2]. While differences in ploidy and large-scale segmental duplication account for some of this variability, differential accumulation (and loss) of mobile genetic elements, especially the class I transposable elements known as long terminal repeat (LTR) retrotransposons, represents an additional and important process through which genome size can vary between individual plant species [3,4]. Plant LTR retrotransposons represent ancient lineages that are ubiquitous in plant genomes [5,6] and can account for >70% of the nuclear DNA of some plant species [4]. Transposition of these elements is via an RNA intermediate, which enables new copies to be synthesized, reverse transcribed and subsequently integrated into host chromosomal DNA. This mode of transposition can result in large-scale genome expansion because each intact and functional element can potentially give rise to numerous daughter copies.

Recent years have witnessed considerable advances in our understanding of LTR retrotransposons. For example, we now know that these elements are tremendously diverse at the sequence level (in plants) with many subgroup lineages existing within the superfamily types *Ty1/copia*-like (Pseudoviridae) and *Ty3/gypsy*-like (Metaviridae) [7-12], that proliferation of these elements can rapidly restructure host genomes [13-16], and that their distribution along chromosome arms can be either dispersed or localized [9,17-19]. Recent work also suggests that LTR retrotransposons (and other major classes of transposable elements) may not exclusively represent selfish or junk DNA as previously supposed [20,21] but that, on occasion, transposable elements may have indeed played a more substantial role in generating evolutionary novelty [22-33].

Despite recent advances, we still know surprisingly little regarding how and when these elements become active and proliferate in natural populations; the vast majority of elements remain transcriptionally and transpositionally quiescent during normal growth and development. Various forms of environmental and/or genomic stress have been hypothesized to influence activation. For example, hybridization between genetically differentiated populations and/or species is one means through which these elements are thought to become active [34-38] although activation and proliferation following hybridization is not observed universally [39,40]. Exposure of plants to biotic and abiotic stresses such as bacterial and viral pathogens, phytophathogenic fungal extracts, wounding, protoplast isolation, and cell culture also has been shown to activate some LTR retrotransposons [41,42]. While biotic and abiotic stressors may represent more universal agents of activation, much of the data supporting this conclusion comes from experiments conducted under unnatural laboratory conditions; the extent to which these same stresses (especially those that occur naturally) have led to activation and proliferation in natural populations remains unknown.

**Figure 1:**
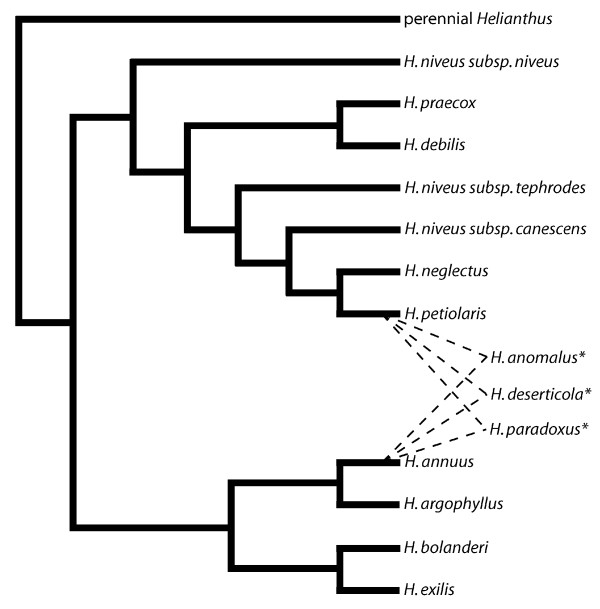
**Evolutionary relationships among annual *Helianthus *species**. Hybrid species are indicated with asterisks. Figure is redrawn from [74] and based on combined nuclear ribosomal and chloroplast DNA data reported in [43].

An especially fascinating case of LTR retrotransposon proliferation in plants involves three annual sunflower species of ancient hybrid origin. These species (*Helianthus anomalus, Helianthus deserticola, and Helianthus paradoxus*) have arisen independently via ancient hybridization events between the same two parental taxa (*Helianthus annuus *and *Helianthus petiolaris*) (Figure 1) [43-45]. Whereas both parental taxa have extensive natural ranges in North America, the three hybrid species are restricted to western and southwestern regions of the United States where they are locally adapted to abiotically extreme environments. The genomes of all three hybrid taxa have experienced spectacular proliferations of *Ty3/gypsy*-like LTR retrotransposons [15,46], resulting in large-scale increases in nuclear DNA content [47]. The evolutionary history of the hybrid species is especially noteworthy given that both hybridization and abiotic stress have been hypothesized to facilitate the activation and proliferation of LTR retrotransposons.

In the current report, we demonstrate that *Ty3/gypsy*-like LTR retrotransposons in sunflower are considerably heterogeneous at the sequence level but yet the same element sublineage has proliferated independently in each hybrid sunflower species. We demonstrate further that the ages of these proliferation events (and thus a lower bound on the hybrid species' origins) can be estimated by examining particular signatures of proliferation found in the hybrid species. Estimates by this method suggest that the hybrid species may be older than previously suggested.

## Results

### Sequence variability of Ty3/gypsy-like retrotransposons in Helianthus hybrid and parental species

The diploid hybrid species possess composite genomes as a result of their hybrid origins [44,48]. The elements that proliferated in the hybrid species' genomes are therefore derived from retrotransposon lineages originally present in the genomes of the parental species *H. annuus *and/or *Helianthus petiolaris*. We surveyed sequence diversity in the two parental and three hybrid *Helianthus *species by amplifying a 520-bp region of the *Ty3/gypsy*-like *rt *domain-encoding region with degenerate primers followed by cloning and sequencing 92 to 108 amplification products per species. Analysis of these sequences revealed considerable diversity in each of the five *Helianthus *species, with pairwise sequence divergences ranging from 0% to 48.7% (*H. annuus*), 0% to 40.5% (*H. petiolaris*), 0% to 39.6% (*H. anomalus*), 0% to 59.9% (*H. deserticola*), and 0% to 36.4% (*H. paradoxus*). Proper reading frames were determined and all sequences translated to assess the frequency of potentially functional copies. Between approximately 20% (*H. petiolaris*) and 42% (*H. deserticola*) of sequences were found to possess indels and/or premature stop codons (Table 1), indicating that a sizable fraction of these elements are no longer likely to be capable of autonomous transposition. These percentages are likely to be underestimates given that we have sequenced only a fraction of the total interior coding region.

**Table 1:** Source of plant material and summary information for sequences of the *rt *domain-encoding region (520 bp) of *Ty3/Gypsy*-like elements

Species	Accession no.^a^	Original collection location	No. of sequences with indels or stop codons	No. of full length sequences without stop codons	Total no. of sequences (no. of sequences in lineage E')
*Helianthus annuus*	PI 468607	Utah: N37.239, W(-113.358)	33	66	99 (29)
*Helianthus petiolaris*	PI 468815	Utah: N37.047, W(-112.530)	21	82	103 (30)
*Helianthus anomalus*	Ames 26095	Utah: N39.744, W(-112.316)	37	71	108 (49)
*Helianthus deserticola*	Ames 26094	Utah: N37.254, W(-113.343)	41	57	98 (47)
*Helianthus paradoxus*	PI 468802	Texas: N30.883, W(-102.983)	19	73	92 (41)

^a^United States Department of Agriculture (USDA) National Plant Germplasm System [76].

### Phylogenetic analyses and sublineage-specific element numerical abundance

**Figure 2:**
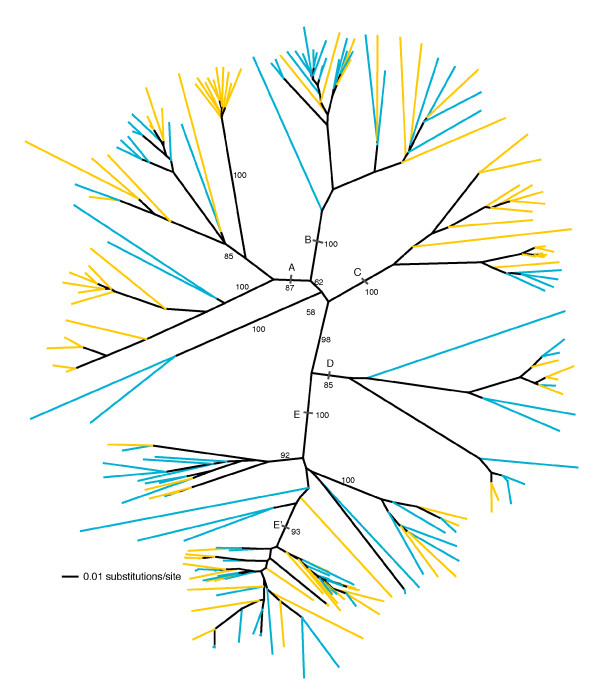
**Unrooted phylogenetic tree of *Ty3/gypsy*-like *rt *sequences (520 bp) isolated from *Helianthus annuus *(blue terminal branches) and *Helianthus petiolaris *(yellow terminal branches)**. Capital letters indicate major lineages (A = *Surge1*, B = *Surge2*, C = *Surge3*, D = *Surge4*, and E = *Surge5*). Bootstrap values (>50%) are shown for branches defining major lineages as well as for deeper internal branches and are based on 1,000 replications. Phylogenetic analysis was conducted using neighbor joining [75].

A phylogenetic analysis of elements derived from both parental species (*H. annuus *and *H. petiolaris*) identified multiple, well supported lineages, with sequences from both *H. annuus *and *H. petiolaris *present in each major lineage (Figure 2). The presence of elements from both parental species in each major lineage indicates that the origins of these *Ty3/gypsy*-like lineages predate the origins of the major clades in which *H. annuus *and *H. petiolaris *reside (see Figure 1). We propose the name *Surge*, (for 'sunflower repetitive gypsy-like elements') for these *Ty3/gypsy*-like retrotransposons in *Helianthus*. In accordance with criteria put forth in [49] addressing family identification and naming of transposable elements, we assign the names *Surge1 *to *Surge5 *for lineages A to E in Figure 2, respectively.

**Figure 3:**
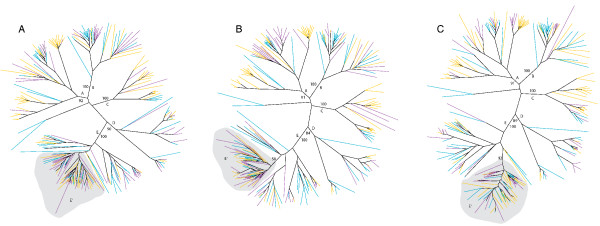
**Unrooted phylogenetic trees of *Ty3/gypsy*-like *rt *sequences (520 bp) isolated from *Helianthus annuus *(blue terminal branches), *Helianthus petiolaris *(yellow terminal branches), and hybrid derivative species *Helianthus anomalus *(a), *Helianthus deserticola *(b), and *Helianthus paradoxus *(c) (sequences from hybrid species are indicated by red terminal branches)**. Capital letters indicate the same reconstructed lineages as defined in Figure 2. The lineage defined by gray shading (lineage E') represents a candidate source lineage for proliferative retrotransposons. Phylogenetic analysis was conducted using neighbor joining [75]. Bootstrap values (>50%) are shown for major identified lineages.

**Figure 4:**
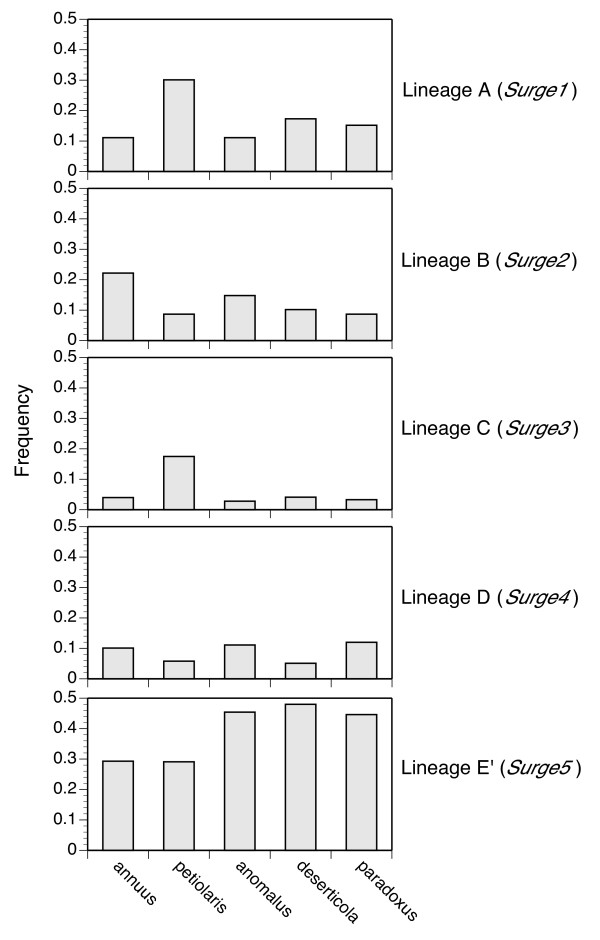
**Lineage-specific (see Figures 2 and 3) frequency of *Ty3/gypsy*-like sequences from the five *Helianthus *species under investigation**.

Phylogenetic analyses on data sets including sequences from both parental species and a single hybrid species (that is, *H. annuus *+*H. petiolaris *+ *H. anomalus; H. annuus *+ *H. petiolaris *+ *H. deserticola*; and *H. annuus *+ *H. petiolaris *+ *H. paradoxus*; Figure 3a–c, respectively), yielded similar results with respect to the distribution of sequences across major identified sublineages and additionally revealed that a single sublineage (shaded gray in Figure 3a–c; lineage E' in Figure 4; Table 1) consistently harbored a higher abundance of sequences derived from the hybrid species' genomes than from the parental species' genomes. This sublineage lies within a larger, well supported major lineage (designated as lineage E, or *Surge5*). This pattern of consistent differential abundance between parental and hybrid species of sequences in lineage E' presumably emerges because elements that are more common (that is to say, have proliferated) in the hybrid species' genomes are more frequently amplified by degenerate polymerase chain reaction (PCR) and have a higher likelihood of being cloned and sequenced. In phylogenetic analyses, these sequences group most closely with related sequences in the parental species' genomes from which they are likely derived. This pattern of consistent differential abundance between hybrid and parental taxa in sublineage E' was not observed for any other *Ty3/gypsy*-like lineage (Figure 4).

### Proliferation events inferred from frequency distributions of divergence values

Signatures of transposable element proliferation in species' genomes also can be characterized through analysis of the distribution of divergence values between pairwise combinations of element sequences [50,51]. This form of analysis relies on the fact that all daughter copies of transpositionally active elements are identical at the time of insertion but subsequently accumulate mutations independently. Peaks in the distribution of divergence values correspond to episodes of transposable element proliferation, with peaks associated with greater divergence representing more ancient proliferation events and peaks associated with lesser divergence representing more recent events.

**Figure 5:**
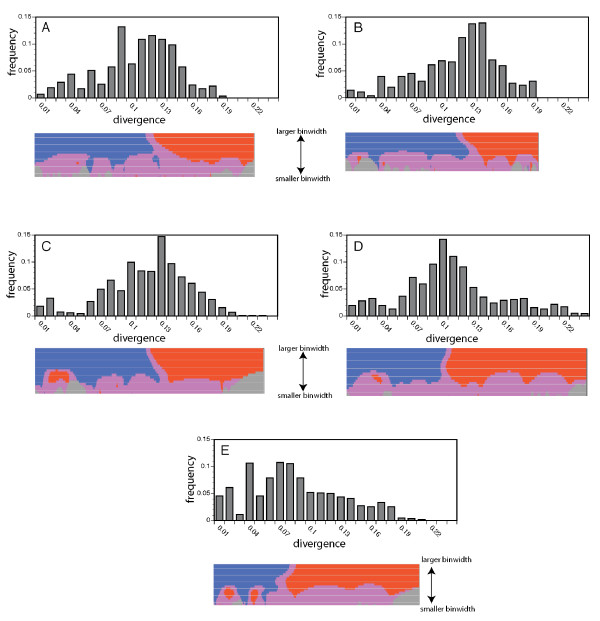
**Distributions of pairwise sequence divergence values demonstrating episodes of retrotransposon proliferation in sublineage E'**. The *x *axis represents sequence divergence; *y *axis represents the frequency of values. **(a) ***Helianthus annuus*; **(b) ***Helianthus petiolaris*; **(c) ***Helianthus anomalus*; **(d) ***Helianthus deserticola*; **(e) ***Helianthus paradoxus*. Below each distribution is a siZer [52] map indicating the strength of support for features (peaks) in the distribution. At a given binwidth, features are detected as significant increases in the slope (indicated by blue) followed by significant decreases in the slope (indicated by red). Purple indicates that the confidence interval for the derivative contains zero. Gray indicates insufficient data for analyses.

Phylogenetic analyses implicate a single *Ty3/gypsy*-like sublineage (sublineage E') as a candidate proliferative source lineage of *Ty3/gypsy*-like retrotransposon amplification in the hybrid species. Distributions of pairwise divergence values for sequences from within this sublineage are depicted in Figure 5a–e for the two parental and three hybrid *Helianthus *species. The program siZer [52] was employed to evaluate these distributions for evidence of significant features (peaks) (see Methods). Analyses of these distributions indicate strong support for a single large peak in the range 0.1 to 0.13 for *H. annuus*, *H. petiolaris*, *H. anomalus*, and *H. deserticola *and at approximately 0.07 for *H. paradoxus*. Strong support for smaller secondary peaks at lower divergence values (0.02 to 0.03) was additionally found for *H. anomalus*, and *H. deserticola*, and strong support for two such additional secondary peaks (at 0.02 and 0.04) was detected for *H. paradoxus*. There was some (albeit much weaker) support of secondary features in the distribution of *H. petiolaris *values, though these were only detected over a very narrow range of binwidths. There was no support for secondary peaks in *H. annuus*. Peaks at lower divergence values suggest recent retrotransposon proliferation events and support our assertion (based on phylogenetic data) that sublineage E' is indeed a proliferative source lineage.

Timeframes for proliferation events indicated by these secondary peaks can be explored given that genome-level mutation rates for *Helianthus *have been estimated. In wild sunflowers, a silent site mutation rate has been estimated at 1.0 × 10^-8 ^substitutions/site/year based on sequence comparisons in a large EST database coupled with fossil calibrations (M. Barker and L. Rieseberg, University of British Columbia, personal communication). It has been suggested, however, that mutation rates for LTR retrotransposons may be approximately twofold higher than silent site mutation rates for protein coding genes [53]. Thus, utilizing a mutation rate of 2.0 × 10^-8 ^to account for elevated sequence evolution of *Ty3/gypsy*-like retrotransposons, proliferation events indicated by peaks at 0.02 to 0.04 divergence are roughly estimated to have occurred some 0.5 to 1 million years ago. Timeframes for proliferation events indicated by more prominent primary peaks were not estimated because these features were found in both the hybrid and parental species. It is thus inferred that proliferations associated with these features predate the origins of the hybrid taxa.

### Phylogenetic relationship of Surge1 to Surge5 elements to other plant Ty3/gypsy-like retrotransposons

**Figure 6:**
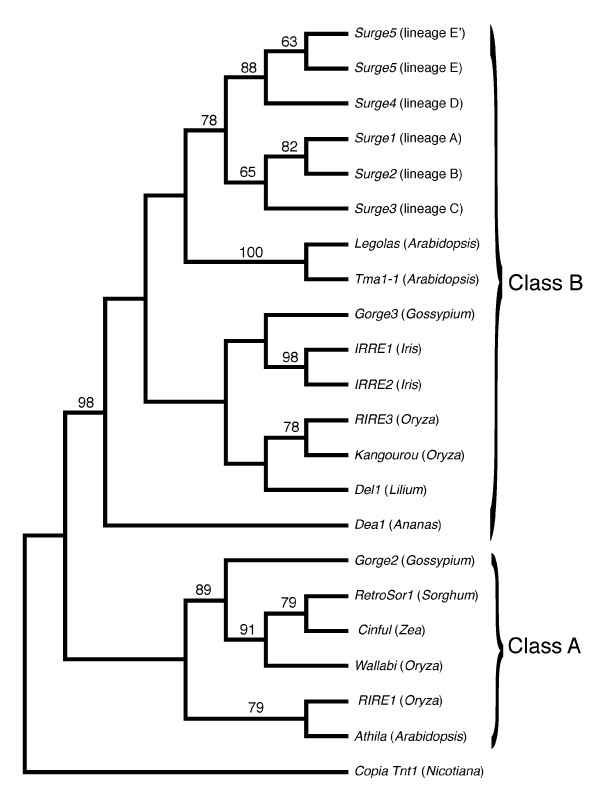
**Phylogenetic relationship of *Surge *elements to other plant *Ty3/Gypsy*-like retrotransposons based on 118 parsimony-informative residues of the *rt *domain**. The *Tnt1 Copia*-like element from *Nicotiana *was used as a phylogenetic outgroup. The alignment of ingroup and outgroup sequences was conducted manually with the aid of retroelement alignments reported in [76]; phylogenetic analysis was conducted using the heuristic search option of maximum parsimony. One of the three most parsimonious trees is reported. Bootstrap values (>50%) are indicated above branches and are based on 5,000 replicates. Class A and class B designations follow those reported in [12]. GenBank accession numbers and/or citations for sequences are: *Legolas *(AC007730), *Tma1-1 *(AAD22339), *Gorge3 *(EU098898), *IRRE1 *(consensus sequence reported in [39]), *IRRE2 *(consensus sequence reported in [39]), *RIRE3 *(AB014738), *Kangourou *(DQ365821), *Del1 *(X13886), *Dea1 *(Y12432), *Gorge2 *(DX404795), *RetroSor1 *(AAD19359), *Cinful *(AAD11615), *Wallabi *(DQ365824), *RIRE1 *(BAA22288), *Athila *(AB005248), *Copia Tnt1 *(P10978).

Evolutionary relationships of *Surge1 *to *Surge5 *elements to other plant *Ty3/gypsy*-like retrotransposons were evaluated by phylogenetic analysis of aligned amino acid sequences of the *rt *domain. A single, full length sequence was randomly selected from each major lineage identified in Figure 2 and included in a phylogenetic analysis with *Ty3/gypsy*-like LTR retrotransposons isolated from the genomes of other plants. Figure 6 depicts one of three most parsimonious trees that differ only in the placement of the *RIRE1*/*Athila *clade relative to two other well supported clades (the first designated as 'class B' and the second consisting of *Gorge2*, *RetroSor1*, *Cinful*, and *Wallabi*). The *Surge *elements form a well supported monophyletic group within the class B *Ty3/gypsy*-like retrotransposons and were most closely related to elements isolated from *Arabidopsis thaliana*.

## Discussion and conclusion

### Ty3/gypsy-like retrotransposon proliferation in Helianthus hybrid taxa

Despite the ubiquity and abundance of LTR retrotransposons in plant genomes, our understanding of the dynamics of their proliferation and the consequences of proliferation events on host species evolution is surprisingly limited. Sunflower species in the genus *Helianthus *provide an excellent group for investigating the possible causes and potential consequences of LTR retrotransposon proliferation in an ecological and evolutionary context. In a previous report [15], we demonstrated that three ancient hybrid sunflower species have independently experienced massive proliferation of *Ty3/gypsy*-like LTR retrotransposons following their origins. The current study examines the dynamics of these proliferation events in light of the known relationships among the sunflower species investigated and the requisite condition that proliferative elements in the hybrid species are necessarily derived from lineages present in one or both parental species.

As is commonly observed in plant genomes, we found *Ty3/gypsy*-like retrotransposons to be considerably diverse at the sequence level, with multiple well supported phylogenetic lineages identified. Particular elements that undergo proliferation, however, are expected to be more abundant in a species' genome, and thus more frequently amplified, cloned, and sequenced via the degenerate PCR methodology employed in this study. Consistent with this expectation, element sequences in the same single sublineage were consistently more abundant numerically in each of the three hybrid species' genomes relative to the genomes of the parental species. This pattern is unlikely to have emerged stochastically via PCR drift given that the same pattern was observed for all hybrid taxa. Moreover, the cloning of degenerate PCR amplification products was conducted on pools of five independent PCR reactions per species, further reducing the likelihood of observing this pattern by chance. This pattern also cannot be attributed to variation in primer sequence specificity across the sunflower species because the degenerate primers used in this study were based on aligned amino acid sequences of several plant species (see Methods), with *H. annuus *(a parental species) as the sunflower representative. Additionally, our interpretation of this phylogenetic signal is corroborated through independent analyses of the frequency spectra of pairwise sequence divergences (Figure 5a–e).

That proliferation of the same sublineage of *Ty3/gypsy*-like retrotransposon has occurred independently in each of the three hybrid sunflower species is of considerable interest, and future work will examine this lineage in greater detail to determine whether transcriptional and/or transpositional activation can be detected in natural and/or greenhouse synthesized hybrids between the parental species *H. annuus *and *H. petiolaris*. It is noteworthy that elements within this sublineage also are fairly abundant in the parental species, indicating past amplification in the parental species as well. Based on limited sampling, however, these elements do not appear to be currently active transcriptionally in either the parental or hybrid taxa (RT-PCR data not shown), a result that lies in contrast to another study examining diversity and abundance of *Ty3/gypsy*-like elements in wild Iris species and their early generation hybrids [39].

Another potentially relevant factor in these proliferation events may be the demographic history of these sunflower hybrid species. Recent work [54] has demonstrated that several categories of transposable elements display differential patterns of distributional abundance and presumed activity among natural populations of *Arabidopsis lyrata *that have and have not experienced historical bottlenecks during postglacial recolonization into new geographical regions. Following arguments put forth previously [55,56] the authors invoke weaker selection against transposable element activity in bottlenecked populations resulting from reductions in effective population sizes and the accompanying increased strength of genetic drift. It is conceivable that similar demographic forces may have acted in the *Helianthus *hybrid species given differences in habitat preferences between the hybrid and parental sunflower species and the founder event-like population structures that may have been associated with the hybrid species' origins.

While this study indicates clear patterns of retrotransposon proliferation events in the genomes of these sunflower hybrid species, some caveats need mention. First, it is unlikely that we have sampled the total *Ty3/gypsy*-like diversity in these *Helianthus *genomes. The sequence variability reported here is limited by the degeneracy of the primers employed. More comprehensive methods for uncovering the full range of retrotransposon subfamily diversity would require genome-level sequencing efforts. For example, by analyzing whole genome shotgun (WGS) libraries, Hawkins *et al*. identified three major subfamilies of *Ty3/gypsy*-like elements in *Gossypium *species, naming them *Gorge1*, *2*, and *3*. Similarly, utilizing available large sequence datasets for *Oryza australiensis*, Piegu *et al*. also characterized three major subfamilies of *Ty3/gypsy*-like elements. The sunflower *Ty3/gypsy*-like elements described in the current study are most closely related to the *Gorge3 *and *Kangourou *subfamilies of elements identified by Hawkins *et al*. and Piegu *et al*., respectively (Figure 6); this suggests that additional *Ty3/gypsy *diversity in *Helianthus *remains uncharacterized. A second caveat of this study, and related to the first, is that we have surveyed sequence variability of the more conserved reverse transcriptase domain-encoding region in the current study whereas our earlier report of proliferation in the hybrid species [15] was based on comparisons among parental and hybrid species of relative abundance (Southern blot) and absolute abundance (quantitative PCR) of the integrase domain-encoding region. Thus, while we assume we have documented proliferation of the same *Ty3/gypsy*-like subfamily in the current and earlier report, we cannot rule out the possibility that we have identified different subfamilies in these two studies and we currently lack the resolution to detect this possibility. This matter can be resolved through additional surveys of sequence variability and by isolating and sequencing the entire protein-coding interior regions for a diversity of these elements. A third caveat is that although the parental taxa that gave rise to the hybrid species are still extant, certain retrotransposon lineages could have been lost from the genomes of one or both parental species over evolutionary time; thus, the genomic composition of modern day *H. annuus *and *H. petiolaris *may differ in some fashion from that of the *H. annuus *and *H. petiolaris *individuals/populations that originally gave rise to the hybrid species.

### Distributions of element divergence values and temporal estimates of proliferation events in the Helianthus diploid hybrid species

Distributions of divergence values for sequences within the candidate proliferative source lineage E' revealed strong evidence of secondary features (peaks) associated with lower values of divergence (Figure 5a–e) in the hybrid species, with no evidence of such peaks in *H. annuus *and only limited evidence of such peaks in *H. petiolaris*. This pattern is exactly that predicted under a scenario of element derepression and proliferation in the diploid hybrid taxa following or associated with their origins. The specific sets of sequences that give rise to these secondary features appear to differ among the three hybrid taxa (data not shown), providing further evidence of independent proliferation events in the three hybrid species. Evidence of peaks associated with lower divergence values in *H. petiolaris *was weak and observed only under a very narrow range of binwidths in analyses with the program siZer [52]. Nonetheless, we cannot rule out recent activity of a lesser scale in this parental species.

Ty3/gypsy-like proliferation events in the hybrid species' genomes offer a unique opportunity to explore the temporal origins of these species given that proliferation events occurring in the hybrid taxa place a lower bound on their birth. Using methods for dating the ages of paralogous sequences within genomes [50,51,57], these proliferation events in the hybrid species are estimated to have occurred between approximately 0.5 to 1 million years ago. These estimates suggest an earlier origin for the hybrid taxa than has been previously suggested based on microsatellite divergence data [58-60], but are largely consistent with more recent revised estimates based on EST sequence divergence data (L. Rieseberg, University of British Columbia, personal communication).

### Retrotransposon proliferation and species evolution

An outstanding question remains how, if at all, transposable element proliferation may have contributed to evolutionary events that took place in this group of sunflowers. Might this represent an example of new species arising via hybridization and concomitant 'reassortments of repetitious DNAs', as envisioned originally by McClintock [61]? Earlier views by other prominent researchers suggested that transposable elements likely represent 'selfish' or 'parasitic' DNA sequences only, and that a major role in evolutionary processes of the host need not be invoked in order to explain their existence [20,21]. More recently, however, several researchers have argued that transposable elements may indeed play a more prominent role in generating evolutionary novelty with potential effects on adaptive evolutionary change [22,23,27,28,30,62-65]. The vast amounts of sequence data now available for several species appear to be supportive of this more recent view, especially with regard to the LTR retrotransposons. For example, genomic sequence data for several model species including *Mus musculus*, *Caenorhabditis elegans*, and *Drosophila melanogaster *indicate that LTR retrotransposons sequences (and fragments thereof) show a higher than expected prevalence of association with certain categories of genes, and that novel gene configurations can arise via new exons or spliced additions to existing exons [24-26]. In addition to generating evolutionary novelty via new (or modified) protein sequences, recent studies also suggest that LTR retrotransposons as well as other types of transposable elements may influence regulatory aspects of host genes [31-33].

Notwithstanding any contribution to evolutionary novelty in this group, it is also intriguing to ponder how these sunflower genomes simply have accommodated massive genomic expansions given the highly mutagenic nature of such large-scale proliferation events [15]. Interestingly, in phylogenetic analyses with other plant *Ty3/gypsy*-like retrotransposons, *Surge *elements group within the 'class B' elements (as described by Martin and Llorens [12]. Elements in this group possess an additional domain (a chromodomain) in their interior coding region that is involved in site-directed integration of elements into heterochromatin [66]. The spatial scale of proliferation in the hybrid species conforms with expectations of site-directed insertion, as fluorescence *in situ *hybridization (FISH) experiments reveal that the vast majority of retrotransposon proliferation in the sunflower hybrid taxa has occurred in pericentromeric regions of chromosomes [67]. The amenable nature of this group of sunflowers to experimental study and the excellent genomic resources now emerging for *Helianthus *should greatly facilitate future work on the likely causes and evolutionary consequences of retrotransposon proliferation in these species.

## Methods

Seeds of all species under investigation were obtained from the United States Department of Agriculture (USDA) National Plant Germplasm System (Table 1). Seeds were germinated in the dark on moist filter paper in Petri dishes and germinated seedlings were then transferred to 10 cm pots and grown in the Kansas State University greenhouses until suitable size for harvesting of plant tissue. DNA was extracted using a DNeasy Plant Mini Kit (Qiagen, Valencia, CA, USA) following the manufacturer's instructions.

Degenerate primers (forward, 5'-GGACCTGCTGGACAAGGGNTWYATHMG-3' and reverse, 5'-CAGGAAGCCCACCTCCCKNWRCCARAA-3') were developed and used to amplify a 520 bp fragment of the *Ty3/gypsy*-like *rt *domain-encoding region from a single plant of each species. Degenerate primers were developed with the web-based program CODEHOP [68] and were based on aligned *Ty3/gypsy*-like reverse transcriptase (*rt*) amino acid sequences from sunflower [69] and the following additional elements: *Dea1, Ananas*; *Del1, Lilium*; and *Legolas, Arabidopsis*. PCR was performed on an MJ Research (Watertown, MA, USA) PTC-100 thermal cycler under the following conditions: 3 min at 94°C, followed by 31 cycles of 30 s at 94°C, 30 s at 45°C, and 60 s at 72°C, and a final extension of 3 min at 72°C. Individual PCR reactions each contained 5 ng DNA, 50 pmol of each primer, 1 unit of *Taq *polymerase, and a final concentration of 30 mM tricine, 50 mM KCl, 2 mM MgCl_2_, and 100 μM of each dinucleotide triphosphate (dNTP). For each sunflower species, five individual 25 μl PCR reactions were performed and pooled for further processing in order to reduce potential effects of PCR drift [70]. Pooled products were gel purified using a QIAquick Gel Extraction Kit (Qiagen, Valencia, CA, USA) and cloned using the pGEM-T Vector System I (Promega, Madison, WI, USA). For each species, between 96 and 109 positive clones were sequenced using the M13 universal sequencing primer on an ABI 3730xl Genetic Analyzer. In a small number of instances two or more sequences obtained from the same species demonstrated 100% identity. Because of the inability to distinguish between a single amplicon variant having been cloned more than two times versus independent amplification of identical elements with different chromosomal insertion points, only a single representative of identical sequences was retained so as not to bias interpretation in subsequent analyses (see Results). Thus, for each species, between 92 and 108 unique sequences were retained for further analysis (Table 1). Sequence alignments were conducted with ClustalW [71] with subsequent manual adjustments. Phylogenetic analyses were conducted in PAUP* v.4.0b10 [72] using the Kimura two-parameter model of sequence evolution. Sequences used in this study have been deposited in GenBank under accession numbers GQ366796–GQ367295.

Evidence of recent retrotransposon proliferation events in the hybrid species was evaluated by examining distributions of pairwise divergence values for sequences derived from a candidate proliferative source lineage as suggested by our phylogenetic analyses. Peaks in the frequency distribution are interpreted as episodic events of transposition, with peaks associated with lower values of divergence representing more recent proliferation events. Pairwise divergence values among sequences were determined using MEGA version 4 [73] under the Kimura two-parameter model of sequence evolution. In order to identify significant features (peaks) in these distributions, we utilized the program siZer [52]. This program evaluates data over a wide range of binwidths and determines statistical support for features (peaks) in a distribution based upon regions of significant slope increase and decrease.

## Authors' contributions

MCU conceived and designed the study. SCS and KMS generated the data. SCS and MCU analyzed the data. MCU wrote the manuscript.
